# Exploration of facilitators and barriers to the regulatory frameworks of dietary and herbal supplements: a scoping review

**DOI:** 10.1186/s40545-022-00447-7

**Published:** 2022-09-05

**Authors:** Jeremy Y. Ng, Minji Kim, Ayush Suri

**Affiliations:** Department of Health Research Methods, Evidence, and Impact, Faculty of Health Sciences, Michael G. DeGroote Centre for Learning and Discovery, McMaster University, Room 2112, 1280 Main Street West, Hamilton, ON L8S 4K1 Canada

**Keywords:** Dietary health supplements, Scoping review, Regulation, Regulatory frameworks, Health policy

## Abstract

**Background:**

Over the last decade, the use of dietary and herbal supplements (DHSs) has expanded greatly across national healthcare settings for the prevention, management and/or treatment of a variety of health issues. Although regulatory policies for DHSs across different countries have been aimed towards evaluating safety and efficacy, performing quality controls, monitoring the manufacturing practices, and encouraging further research, significant safety issues have emerged from inappropriate regulatory classification, lack of suitable quality controls, and inadequate testing and labeling of these products. Therefore, the purpose of this scoping review was to identify facilitators and barriers to DHS regulation across different countries.

**Methods:**

A scoping review was conducted and informed by the five-stage methodological framework proposed by Arksey and O’Malley and further enhanced by Levac et al. MEDLINE, EMBASE, AMED, and PsycINFO databases were systematically searched for eligible articles from database inception to September 29, 2020. Studies analyzing DHS regulatory frameworks were eligible for review. Relevant data from eligible articles were extracted and categorized into themes to provide a descriptive overview of the literature.

**Results:**

Searches generated 4314 results, 1800 of which were duplicates, followed by 2472 that were excluded after screening the titles and abstracts of these articles. Of the remaining 42 full-texts, 15 eligible articles were included in this review. Themes identified include: (1) increased financial and human resources encouraging knowledge expansion as a facilitator to DHS regulation; (2) variances in DHS classification and regulatory requirements across countries as a barrier to DHS regulation, and (3) collaboration between various stakeholders (experts, policymakers, representatives of regulatory bodies, product companies and research institutions) facilitating DHS regulation.

**Conclusion:**

The present scoping review identified facilitators and barriers to DHS regulation across different countries. We highlight that safety assessments of DHSs continue to be inadequate, and emerging technologies could potentially play a significant role in establishing common reference standards of herbal materials and products between regulatory agencies. Regulatory harmonization, increased scientific research, and collaboration could improve regulations globally through appropriate categorization and safe application of DHSs.

## Background

Over 80% of the global population relies on dietary and herbal supplements (DHSs) to supplement their healthcare needs and prevent common ailments [1, 2]. DHSs include vitamins and minerals, herbal supplements, enzymes, amino acids, and tissue from organs or glands [3]. They are marketed in various forms such as tablets, powders, and tinctures, and are used for a variety of reasons such as to supplement diets, help with medical conditions, boost energy, or improve quality of sleep [4]. Similarly, herbal supplements contain one or more herbs that can be any form of a plant or plant product to improve overall health [3, 5].

There are different terms used in reference to DHSs across the literature, including natural health products (NHPs), herbal medicines (HMs), and indigenous natural products (INPs), which can be used in traditional and complementary medicine (T&CM) systems, such as traditional Chinese medicine (TCM). We refer to these terms as DHSs for the purpose of this review, but acknowledge that there are inherent differences between these terms and their regulations within a specific jurisdiction and that these products may overlap across categories between different countries. Therefore, it is important to define these variances between international categorizations: NHPs, like DHSs, include vitamins and mineral supplements, homeopathic medicines, herbal therapies, and also comprise other traditional medicines subclasses such as Ayurveda, and Native North American medicines [6]; traditional medicine is a system based on the knowledge, skills, and practices belonging to different cultures that may include the use of DHSs for the maintenance of health [7]; HMs are one type of dietary supplement obtained from vegetable, fungal or algae sources as active raw materials with therapeutic or other human health benefits [7]; TCM includes DHSs as a subset of herbal remedies and pharmacologically active substances grown throughout China, but it also includes acupuncture, moxibustion, massage, food therapy, and physical exercise [8]; INPs grown in Southern Africa are categorized as herbs and botanicals (a modality of DHSs) and mainly comprise plant products containing active herbal components that can be used as ingredients in medicinal preparations [9].

The general perception of DHSs being safe because they are more “natural” when compared to prescription medications is often misleading, as some DHSs have been shown to cause various adverse reactions, some of which may result in serious injuries and life-threatening conditions [1, 10–13]. Significant safety issues have also resulted from inappropriate regulatory classifications, a lack of suitable quality controls, and inadequate testing and labeling of these products. For example, countries that classify traditional and HM as foods or dietary supplements do not require evidence of safety and efficacy before being marketed or require fewer rigorous product quality tests [1]. In the case of the United States, products directly classified under the dietary supplement pathway are not fully assessed before marketing, whereas products categorized as botanical drugs often require stricter scientific evaluation by the government prior to advertising, selling, and/or delivering DHSs to consumers [3, 14]. On the other hand, in Canada, DHSs are referred to as NHPs, and the registration procedure of DHSs is regulated by the Natural and Non-Prescription Health Product Directorate, a branch of Health Canada [15]. NHPs are licensed through two different pathways: (1) NHPs making modern health claims or (2) NHPs claiming to be used as traditional medicines [15].

The nature of regulatory policies for DHSs across different countries has been aimed towards evaluating their safety and efficacy, performing quality controls of the raw materials used, monitoring the manufacturing practices, and encouraging more research to expand DHS knowledge within national drug authorities [10, 16, 17]. For example, the Therapeutic Goods Association of the Australian government established a pharmacovigilance program to assess adverse events reported by complementary medicine consumers, healthcare professionals, and international medical and scientific experts on advisory boards [16]. When compared to countries such as France, Brazil, and Austria, that have established laws regarding DHS registration, Kuwait and Bahrain lack a well-defined regulatory system as they primarily import DHSs from other countries [18]. Moreover, in the United Kingdom, many unlicensed herbal remedies reached consumers without meeting safety or quality standards until 2010 [19]. Subsequently, in 2011, the European Union established a simplified licensing system requiring a product license on all HMs to validate product efficacy, safety, and quality, before being manufactured [19, 20]. To our knowledge, a research gap exists due to the fact that no study has yet systematically identified and summarized research which evaluates the facilitators and barriers to DHS regulation in a global context, thus making this the purpose of the present review.

## Methods

### Approach

A scoping review was conducted to identify the facilitators and barriers to DHS regulation. It was informed by Arksey and O’Malley’s five-stage scoping review framework and further enhanced by Levac et al. [21, 22]. The methodology consists of five stages: (1) identifying the research question, (2) identifying relevant studies, (3) selecting the studies, (4) charting the data, and (5) collating, summarizing, and reporting the results. This method required searching for and assessing the available literature on a given topic to identify the characteristics of eligible articles, summarize their contents, and highlight knowledge gaps.

### Step 1: identifying the research question

The research question was as follows: What barriers and facilitators to the regulation of DHSs can be identified by studies evaluating policies and regulatory frameworks across different countries? For this study, we defined a “facilitator” as any factor that allows or promotes the implementation of DHS regulations. We defined a “barrier” as any factor that prevents or hinders the implementation of DHS regulations.

### Step 2: finding relevant studies

Following a preliminary scan of the literature, we conducted systematic searches on MEDLINE, EMBASE, AMED, and PsycINFO on September 30, 2020 from database inception until September 29, 2020. The search strategy (Table 1) included indexed headings and terms used in the literature to refer to DHS regulation. The reference lists of relevant reviews were also searched for any additional eligible studies.

**Table 1:**
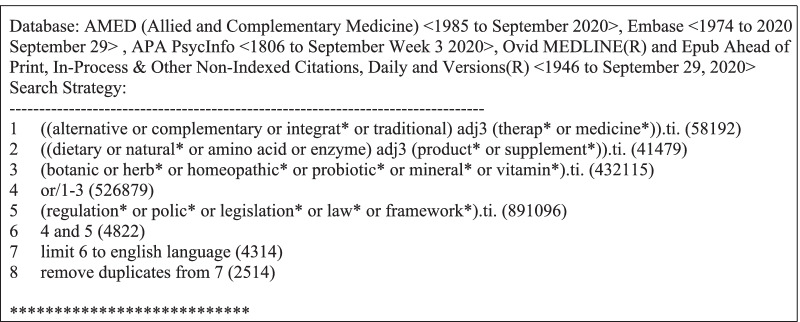
OVID search strategy executed September 30, 2020

### Step 3: selecting the studies

Preliminary searches indicated that evaluative studies have been conducted within this subject area, albeit the volume of literature appeared sparse. We only included articles that discussed the DHS framework of one or more countries. For studies evaluating the regulation of non-DHSs, MK and AS examined what products were in question. The studies were deemed eligible if information pertaining to at least one ingredient of DHSs was discussed. While reviews themselves were excluded, we hand-searched their reference lists for eligible articles. All other types of articles such as commentaries, editorials, or conference abstracts were ineligible. Studies that exclusively evaluated regulatory frameworks of non-DHSs (i.e., complementary and alternative medicines such as acupuncture or chiropractic) were excluded. Studies exclusively evaluating the regulatory framework surrounding cannabis were also excluded as it is commonly regulated separately from DHSs; this work is instead presented in a separate review [23]. All retrieved articles were pilot-screened for eligibility by MK and AS independently and in duplicate, based on the title and abstract, followed by the screening of the full-texts of the articles. After every round of independent screening, all three authors met to discuss and resolve any conflicts or discrepancies.

### Step 4: charting the data

Articles that met the inclusion criteria were critically reviewed using the descriptive–analytical narrative method. The following items from each eligible article were extracted and charted: first author and year; article title; the country where the study was conducted; the countries discussed in the article in relation to DHS regulation; study aim; conclusion; participants or document type/level of policy-making; types of evidence; summary of description of methods; facilitators to the implementation of DHS regulations; and barriers to the implementation of DHS regulations. Data extraction was conducted independently and in duplicate by MK and AS. Following this, all three authors met to discuss and resolve any discrepancies.

### Step 5: collating, summarizing, and reporting the results

All eligible articles were reviewed by MK and AS. Data were then analyzed and organized in a tabular format to provide a descriptive overview of emerging and prevalent themes across the literature. This descriptive data were then categorized into distinct themes by MK and AS. JYN identified commonalities between themes and organized them under overarching thematic categories, and all authors subsequently presented the findings under each corresponding theme.

## Results

**Fig. 1:**
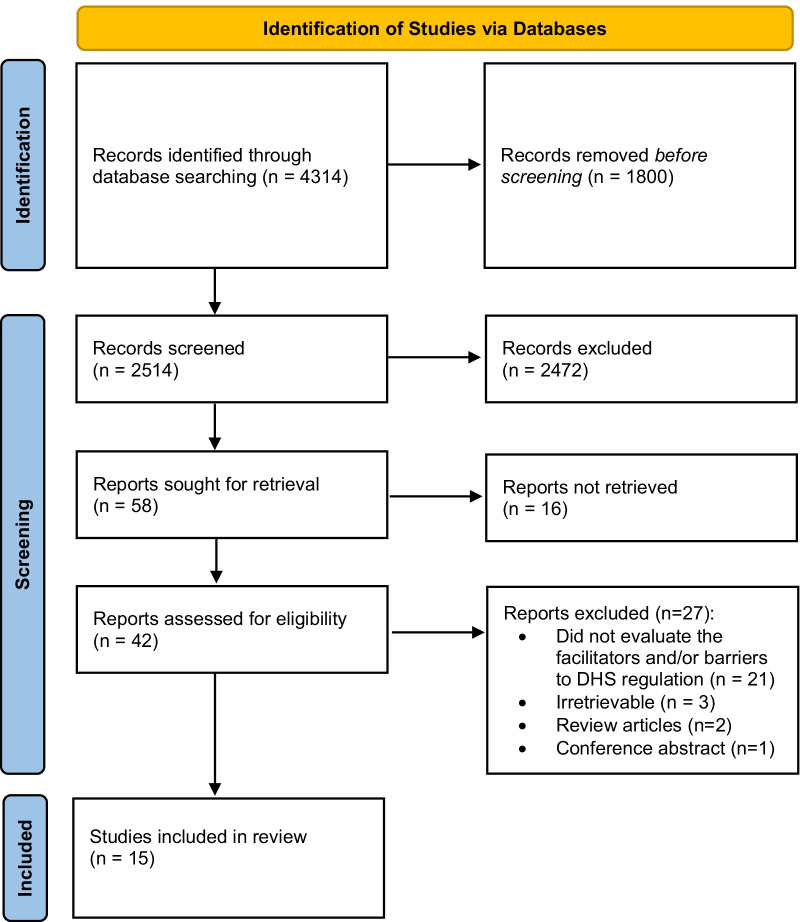
PRISMA diagram

Searches generated 4314 results, 1800 of which were removed as duplicates. Of the remaining items, 2472 were excluded based on title/abstract screening, leaving 42 full-text articles to be further reviewed. Of these, 27 articles were deemed ineligible because they did not evaluate the facilitators and/or barriers to DHS regulation (*n* = 21), were irretrievable (*n* = 3), were review articles (*n* = 2), or was a conference abstract (*n* = 1), resulting in a total of 15 eligible articles that were included in this scoping review. A PRISMA diagram can be found in Fig. 1.

### Eligible article characteristics

Eligible articles were published between 2001 and 2019, and were conducted in Brazil (*n* = 4), Canada (*n* = 4), India (*n* = 1), Lebanon (*n* = 1), Namibia (*n* = 1), Nigeria (*n* = 1), and the United Kingdom (*n* = 1). Additionally, one study originated from both the United States and Ghana, and another study originated from both Bahrain and Kuwait. The 15 eligible studies had various aims ranging from evaluating current international DHS regulations to assessing propositions of new regulatory frameworks. Additionally, interviews (*n* = 7) and surveys (*n* = 5) were the most common methods utilized to gather qualitative data about the current regulatory framework of DHSs in each country and to explore the different perspectives of stakeholders involved. Comprehensive details of the eligible articles can be found in Table 2. Additionally, a detailed summary of policy-making levels and the facilitators and barriers to DHS regulations is located in Table 3.

**Table 2:** Eligible article characteristics (*n* = 15)

First author and year	Article title	Country where study was conducted	Countries discussed	Study aim	Conclusion
Alostad et al. 2019 [18]	A qualitative exploration of Bahrain and Kuwait herbal medicine registration systems: policy implementation and readiness to change	Bahrain and Kuwait	Bahrain and Kuwait	To evaluate the formation and application of recognized herbal medicine registration programs in Bahrain. This was done to formulate recommendations while analyzing the preparedness for implementing various herbal medicine categorization regulations in Kuwait.	By conducting a sound evaluation of the Bahraini drug regulatory authority, the study was able to shed light on current weaknesses of Kuwait’s unpolished HM system. It provides recommendations and demonstrates that the possibility of implementing new changes was high and how these learnings can aid other countries in improving their HMs policy structure overall.
Carvalho et al. 2018 [32]	The Brazilian market of herbal medicinal products and the impacts of the new legislation on traditional medicines	Brazil	Brazil	The purpose was to evaluate the current conditions of herbal medicinal products licensed in Brazil and carry out a comparison with information collected in both 2008 and 2011. This was conducted to further address the progression of herbal licensed products in the country and the consequent ramifications of any new legislation being applied.	The weak points discovered suggest there exists a critical regulatory and manufacturing obstacle in the country due to various reasons. The primary reason being the presence of an increased range of supplement products and markets. Future studies should aim to mitigate these challenges in order to protect consumers’ health. It is also important to note that certain gaps within this regulatory work have not been completely resolved.
Molin et al. 2019 [18]	Regulatory framework for dietary supplements and the public health challenge	Brazil	Brazil	To further assess the proposition of new regulatory frameworks being developed for DHSs in Brazil. Their existing representation and obstacles posed by new legislation were also examined.	The Indigenous Plant Task Team serves as a predominant governing association for INPs in Namibia that provides a well-structured and multi-stakeholder online platform for resource capitalization and stimulation as well as knowledge exchange. Its processes influenced the development of many actions in the segment of policy production and execution.
Ndeinoma et al. 2018 [9]	The Governance of Indigenous Natural Products in Namibia: a policy network analysis	Namibia	Namibia	To discern the numerous governing bodies responsible for monitoring and coordinating the administration of INPs. The aim also included examining the physical relationships between different actors of this industry and the overarching effects they can have on the natural products’ regulation procedure.	The amount of herbal medicinal products approved in Brazil is small in comparison to other countries. Brazil also hosts an increased ratio of non-native medicinal plant species in terms of supplements’ categorization. The number of sellers in the herbal medicinal products markets has decreased from the past few years. However, the market value has risen, indicating that the profitability should be exploited in the years to come.
Alostad et al. 2018 [28]	International comparison of five herbal medicine registration systems to inform regulation development: United Kingdom, Germany, United States of America, United Arab Emirates and Kingdom of Bahrain	United Kingdom	Kuwait, United Kingdom, Germany, and United States of America, United Arab Emirates and Kingdom of Bahrain	To implement a regulation methodology for HMs in Kuwait which is known for only importing these products. This research report assessed the similarities and differences between each of the five countries being studied: United Kingdom, Germany, United States of America, United Arab Emirates and Kingdom of Bahrain.	As a result of the varying definitions in other countries, it has become vital for Brazil to precisely articulate what comprises an HM and prohibit any misinterpretations. Furthermore, it seems that many HMs escape a comprehensive review and are rather portrayed as dietary supplements in some areas across the globe. This is a matter of concern when establishing registration policies in Kuwait, which usually imports such products and aims to evaluate these considering their governing context in their native country.
Appiah et al. 2018 [25]	Identifying strengths and weaknesses of the integration of biomedical and herbal medicine units in Ghana using the World Health Organization’s Health Systems Framework: a qualitative study	USA, Ghana	Ghana	To recognize the strengths and weaknesses of the government-led amalgamation of HMs into the current biomedical methodology in Ghana. This involved using the Health Systems Framework, created by the World Health Organization.	The intertwined relationship between the two healthcare systems depicted as biomedicine and herbal medicine has been critiqued by researchers for their respective challenges. The combination of the two frameworks can expose a variety of research opportunities within this field. Additional training for traditional health practitioners and clinicians in herbal medicine can pave the way for augmented coordination between the two units. Lastly, robust advocacy practices and public exposure is required to raise awareness and educate the general population on the homogenization and employment of the services offered.
da Justa Neves et al. 2015 [34]	Dietary supplements: international legal framework and adulteration profiles, and characteristics of products on the Brazilian clandestine market	Brazil	Brazil, United States, European Union	Assess the existing legislative framework on dietary supplements in the United States, the European Union and Brazil. The study also aimed to identify the description of adulterated and/or unconventional supplements within these markets.	There are varying levels of strictness between the nations being examined with the United States legislation exhibited as being more permissive, Brazil being more restrictive, and the European Union somewhere in the middle. However, there has not been a direct relationship established between the countries’ legal frameworks and its efficacy in preventing the sale of contaminated products. With rising notification and alerts from the US Food and Drug Administration and increased trend in data from the Brazilian Federal Police Department, it has been illustrated that the problem can be attributed to a rise in the contamination issue or an improvement of detection mechanisms. Next steps include educating others about possible risks associated with consuming adulterated or eccentric products and their impact on human health.
Carvalho et al. 2014 [26]	Regulation of herbal medicines in Brazil	Brazil	Brazil	Rich Brazilian biodiversity, for the Brazilian population, is known for being a reliable source of medicinal plants and traditional herbal supplements’ knowledge. In order to enhance the existing regulatory methods, ANVISA has decided to modify its legislation. This study envisions addressing these new Brazilian principles for the regulation of HMs.	Brazil has been modifying the legislative structures for HMs from the past few years. This change was made to synchronize with global HM definition and requirements incorporating the unique facets of the Brazilian HM industry. This reinforces safe access and appropriate consumption of herbal medicinal products and similar products by the public.
Awodele et al. 2014 [7]	Traditional medicine policy and regulation in Nigeria: an index of herbal medicine safety	Nigeria	Nigeria	To examine the current regulatory guidelines surrounding HMs in Nigeria. The aim of this study is also to employ this information and form a country-wide safety index for HMs.	There exists an urgent need for the Federal Ministry of Health to coordinate differing perceptions on conventional medicine and policy-making. This study was able to record this through workshops and collaboration activities on traditional medicines. The suggested solutions have been projected to remedy some of the challenges being faced and can ensure adequate safety and regulation measures of HMs in Nigeria.
Sahoo et al. 2013 [24]	Herbal drug regulation and commercialization: an Indian industry perspective	India	India	To identify the restrictions the Indian government and other constitutional institutions face in regard to manufacturing, commercialization and surveillance/supervision of traditional herbal medicinal products and drugs.	Inauguration of more robust guidelines on quality control and assurance elements as well as formulating marker-dependent procedures are critically needed to create a safe, and reliable representation of HMs in India. Coordination between legislative systems is important to lessen the impediments across different countries. Elevating population pressure for effective, collaborative and equivalent healthcare causes AYUSH (Ayurveda) to analyze and advance in research and development, clinical medicine, herbal medicinal products, to customarily amplify regulatory mechanisms.
Walji et al. 2013 [29]	Governance of natural health products regulation: an iterative process	Canada	Canada	To understand the contextual factors and major challenges involved in implementing regulation of NHPs in Canada.	Health Canada’s experience with implementing regulation of NHPs highlights several challenges including time to process applications, issues with defining the products and labeling. However, there is a need to monitor and survey any adverse effects in both passive and active manner.
Alameddine et al. 2011 [27]	Stakeholders’ perspectives on the regulation and integration of complementary and alternative medicine products in Lebanon: a qualitative study	Lebanon	Lebanon	To explore the regulation and integration of complementary and alternative medicine products in Lebanon, as an example of an Eastern Mediterranean Region country, by consulting stakeholder feedback on the barriers for proper regulation.	The current regulation of complementary and alternative medicine products in Lebanon, which is primarily done through the Ministry of Health, does not sufficiently ensure public safety nor support the integration of complementary and alternative medicine to its healthcare system. Therefore, there is a need for political resolve and cooperation from all the stakeholders for proper regulatory frameworks to be implemented.
Moss et al. 2007 [31]	The professionalization of Western herbalists: response to new product regulations in Canada	Canada	Canada	To assess Canadian Western herbalist leaders’ responses to the new Canadian NHP regulations, showing insight into the increasing organization of this healthcare profession as a result of new healthcare policy.	The extent to which Western herbalists have progressed with the professionalization process and whether this was due to the NHP regulations is unclear and will need to be explored. Western herbalists seem dedicated to continue with their practice to professionalization, but this progression will be affected by internal and external factors, such as cohesiveness of membership and barriers put up by other healthcare groups, respectively. It will be difficult for them to achieve state-sanctioned provincial regulation across Canada.
Laeeque et al. 2006 [6]	The Canadian NHP regulations: industry perceptions and compliance factors	Canada	Canada	To determine which individuals are in compliance with Canada’s new NHP regulations within the interview sample, explore factors that may affect this compliance, as well as to examine the industry’s response to the regulations.	The exit of smaller firms as a result of the Canadian NHP regulations may lead to industry consolidation. Exploration of Canada’s NHP regulations and the industry’s response can potentially inform other countries who plan to implement regulations for their NHPs.
Moss et al. 2006 [30]	New Canadian natural health product regulations: a qualitative study of how complementary and alternative medicine practitioners perceive they will be impacted	Canada	Canada	To investigate the perspectives of practitioners of complementary and alternative medicine with regard to new regulation and its effect on their practices and their relationships with patients and consumers.	There were concerns surrounding the new NHP regulations and their effects on accessibility to the products, which they need in order to practice effectively. Specifically, naturopaths, TCM practitioners, homeopaths, and Western herbalists were concerned. The naturopathic leaders in particular worried that their roles as healthcare professionals may be limited due to NHPs now being defined as over the counter products.

Abbreviations: *ANVISA* National Health Surveillance Agency of Brazil,* DHS* dietary and herbal supplement,* HM* herbal medicine,* INP* indigenous natural product,* NHP* natural health product,* TCM* traditional Chinese medicine

**Table 3:** Summary of participants or document type/level of policy-making, methods, types of evidence, facilitators and barriers

First author and year	Participants or document type/level of policy-making	Types of evidence	Summary of description of methods	Facilitators to the implementation of DHS regulations	Barriers to the implementation of DHS regulations
Alostad et al. 2019 [18]	The main participants that were identified and interviewed included senior and middle managers employed in the registration of HMs in Bahrain and Kuwait drug regulatory authority. Moreover, scientific reviewers who were also involved in the registration process were selected and took part in observations (in regard to how HMs are currently registered and classified in the National Health Regulatory Agency after the implementation of policy and interviews. The managers identified were primary decision-makers of policies impacting HM registration. On the contrary, the scientific reviewers were employees who were responsible for applying policies concerning HM categorization and conducting detailed analysis on quality control.	Comprehensive field notes were written down during the three major segments of HM registration procedure which include the regulatory review and requirements as well as approximate schedules for important breakthroughs in the review methodology. Furthermore, legislative documents relating to HM registration requirements and its guidelines, along with ministerial decrees were also analyzed.	Data were collected in Bahrain, employing a sequential study format. The preparedness was consecutively measured in Kuwait for implementation purposes. Using the policy triangle model proposed by Walt and Gilson was employed. Then, actors, study background and process elements were identified. Data sources were examined for information regarding legislative policies and any direct information about HM registration procedures. Semi-structured interviews were conducted with twenty three officials (8 officials from the Bahraini drug regulatory authority; 5 reviewers and 3 managers, 15 officials from the Kuwaiti drug regulatory authority; 9 reviewers and 6 managers). These actors were all involved in the HM registration processes. Lastly, this data was analyzed using thematic analyses and findings positioned employing the aforementioned model.	Main facilitators included the increased teamwork between varying parties such as the National Health Regulatory Agency officials and external experts. There existed very open lines of communications, sort of the open-door-policy, allowing both groups to expand their knowledge base in regard to policy development. Furthermore, there was also support received from the government, especially the Supreme Council of Health which allowed the regulations to come in a smooth and quick manner. In addition, there was not a need for supplemental training as all reviewers responsible for policy implementation were experts and had experience in the production process. The old reviewers taught the new reviewers everything such as how to use the guideline and why it is important that they use it.	One major barrier includes the variability of how a HM is defined across the world. For instance, the United States categorized it as a food supplement, whereas the United Kingdom licenses it as something altogether. Kuwait imports HM from these two countries, thereby, making the challenge to classify HMs appropriately that much more enormous. There has also been increased resistance from agents to comply with new frameworks being developed from a social and political perspective. It has taken a long time for them to adapt as sometimes these groups are not even aware of the current and updated regulations. Continuous change in HMs regulations: this is very problematic because every country has its own method of assessment and their own classification system, with the same herb you find it banned in one country, but in another country it is classified as a health product.
Carvalho et al. 2018 [32]	Policy-making was conducted on a country/national level. Governmental documents like drug registration documents and license papers of HMs were analyzed. Additional documents included evidence from different pharmaceutical laboratories as well as physician's prescriptions. These prescriptions were obtained in order to differentiate HMs sold under prescription from those categorized as nonprescription.	The research was carried out with ANVISA database.	Information from ANVISA was placed in an Excel spreadsheet. It was categorized under the following columns: commercial name, botanical name, single or combined herbal medicine, over-the-counter or prescription, licensing holding company, business location, licensing validity and therapeutic category. Then, a survey was performed on the notification platform of ANVISA to ensure viable notifications are presented for HM products.	Financial endorsement systems were provided continually for research, development and production purposes in the field of HM. These included funds from the Ministry of Health and other governmental agencies for research and production of herbal medicinal products. A unique notification system was also established for herbal medicinal products permitting quick product release and an expanded repository of drug data on the Brazilian Herbal Formulary. Newer regulations (e.g., on Brazilian biodiversity) have prompted the elevation of access to genetic heritage. Previously, there were multiple steps involved to obtain permission for HM studies, however, the new law only allows new applications for new products after sufficient and reliable market research, making the process more efficient. Brazil also contains a large population that is “all-ears” to HM and natural products being used for therapeutic purposes.	There are not that many professionals who specialize in the field of HM, especially those which require advanced education and knowledge on biopharmaceutical techniques. There has also been a reduced amount of herbal medicinal products available which can be attributed to National Active Pharmaceutical Ingredients suppliers who are unable to comply with the standard production practices, causing Brazil to import the majority of its HMs. Even with ANVISA organizing several workshops and technical meetings, these were not enough to improve the number of licensed herbal medicinal products. Many pharmaceutical companies do not attend them or use these resources and, as an example, there exists medicinal plants that do not have any licensed herbal medicinal products.
Molin et al. 2019 [18]	Dietary supplement samples were previously placed into different categories based on the efficacy ratings given by governmental websites. However, researchers evaluated this information and created a new categorization criteria for them. The allowed claims are constituted in ANVISA RDC No. 242 and 243/2018. Following, the claims submitted for the key ingredients were collated with recommendations made by international bodies and consortiums such as the Academy of Nutrition and Dietetics, Dietitians of Canada as well as scientific articles.	The study analyzed various dietary supplement selections using the appropriate legislative procedures for this group of products [(I) Food for athletes; (II) Vitamin and/or mineral supplements; (III) New foods and/or new ingredients; (IV) Food of functional and/or health property; (V) Specific medications; and (VI) Herbal medicines]. The e-commerce stores examined in this study (*n* = 7) were all Brazilian. Sample prices within these stores ranged from R$ 19.50 to R$ 244.45 With current budget limitations, along with specifications to portray the sample as a sample of interest, only 44 samples could be obtained. These samples were acquired through mail.	A qualitative, observational and descriptive study was conducted using Google as the primary tool of dietary supplements sold in Brazilian online stores. Product ingredients, side-effects, outlying commercial claims were all assessed. This was to promote and shed light on how existing regulations can be further improved. Effects and outcomes related to specific ingredients as described by manufacturers were compared with theoretical information presented in scientific literature.	It has been noted how a great deal of the population have an increased distrust in modern medicine and are relying more on natural (especially HMs) and alternative therapies for medical illnesses. This is because many associate the word “natural” with a healthy human being that uses safe practices derived from medicinal plants to better his lifestyle and cure diseases. Moreover, more and more individuals are attracted towards the idea of using such natural products as “self-medication” in order to have better control over their own health.	There exist commercial appeals used by business professionals to attract buyers in purchasing their products. Not only that, but the sellers promote a fake image that the products being sold are highly effective, without backing them up with any sort of research. Advertising rules are not being followed by suppliers to the same extent as a result of inconsistency in the inspections carried out by regulatory agencies. The rise of digital transformation and e-commerce platforms have allowed for a growth in the number of dietary supplements available on the market, as well which indirectly contributes to the inefficiency of the inspection of these products.
Ndeinoma et al. 2018 [9]	Creation of Indigenous Plant Task Team, chief governing body for resource mobilization and knowledge exchange. The purpose of its formulation was to have a supervisory group for INPs in Namibia that aspires to contribute a properly enunciated, multi-stakeholder medium for resource mobilization and information sharing.	In the study, two modes were used for information acquisition: open-ended interviews and a questionnaire. These helped to create a governance model from the information provided by the subjects. Then, official documents were analyzed precisely which included workshop proceedings, and policy documents. In order to identify the varying networks and types of activities during interviews, legislative proceedings for multiple governance bodies were also assessed.	Residents that originated from communal areas were used. They resided in areas where varying INPs are grown. A questionnaire was also employed which contained a list of INP stakeholders, and the participants had to choose with whom they had previously collaborated with. Ties between potential actors and firms could be established through this. The data were also going to be used to formulate a governance model which illustrates trends of actor relations within the INP field in Namibia. This was carried out to get a better picture on possible effects of such interminglings on INP policy-making.	Creation of Indigenous Plant Task Team, chief governing body for resource mobilization and knowledge exchange. The purpose of its formulation was to have a supervisory group for INPs in Namibia that aspires to contribute a properly enunciated, multi-stakeholder medium for resource mobilization and information sharing.	The roles and functions of the Indigenous Plant Task Team were not understood well enough by the general public and even some of its own members. There has been a lack of participation and engagement by representatives of traders or harvesters in meetings and multi-stakeholder forums for INP. Furthermore, the representatives of representatives from the formal state bureaucracies, non-governmental organizations and academic institutes outnumber the representatives of locally based primary producers. The policy network may limit the number of members that can attend certain meetings leading to this issue.
Alostad et al. 2018 [28]	Authorized government law documents signifying HM registrations were used. These papers were obtained from the drug regulatory authority websites of the five countries.	Official documents were analyzed which were acquired from each countries’ governmental and drug regulatory authority database: United Kingdom Medicines and Healthcare Products Regulatory Agency; Federal Institute for Drugs and Medical Devices Germany; United States Food and Drug Administration; Department for Pharmacy and Drug Control United Arab Emirates; and Bahrain National Health Regulatory Authority.	A qualitative study was performed including five countries where HMs were picked: the United Kingdom, Germany and the United States, United Arab Emirates and Kingdom of Bahrain. Documentary analysis followed by carefully evaluating official law documents of the nations’ drug regulatory authority platforms. This data enclosed registration guidelines, and HM definition and classification which were tabulated using Excel. Similarities and differences were compared and assembled.	Improved regulations have been able to increase both the access and variety of HMs being available for customers. Lack of adequate evaluation can affect consumer safety. Since many HMs are imported in Kuwait, a key challenge is the variable definition an HM carries across global drug regulatory authorities. This expedites the inconsistency in Kuwaiti Drug Regulatory Authority where some products such as imported dietary supplements from the United States, are registered under units with insufficient assessment measures. A countermeasure to this would be the adoption of a harmonized system and having a single unified definition of an HM. This would create standardized regulation procedures that will permit these products to be sold in a clear and safe fashion across the globe. Numerous dietary products escape robust review methods since they are classified under dietary supplements instead, raising another concern. Such challenges need to be considered and acted upon by updating and modifying registration systems in countries like Kuwait.	The lack of registration and assessment of herbal products can be a major disadvantage as the assessment of safety has become more challenging in the United States due to the type of regulation used. Moreover, in the process of improving HM legislation, the Kuwait Drug and Food Control Administration has to consider precisely on how to better regulate products that are arriving from countries where such regulations are obscure or very lax.
Appiah et al. 2018 [25]	Individuals performing or having an interest in HM research were interviewed for this study. A grand total of 25 participants were involved. Of these, 18 were from the Faculty of Pharmacy and Pharmaceutical Sciences, two were from the School of Public Health, one was from the College of Science and one was from the College of Arts and Social Sciences. The aforementioned sub schools and faculties all fall under Kwame Nkrumah University of Science and Technology. Lastly, one informant was interviewed from the Centre for Plant Medicine Research and one participant each from two of the piloted hospitals.	Those having a fundamental learning interest in performing meaningful and scientific research in the realm of HM were used as subjects. These individuals were interviewed to garner information about their opinions in relation to integrating herbal medicine with biomedicine in hospitals in Ghana. Detailed notes on the main ideas were taken throughout the interview by researchers, and speeches were made into transcripts.	This is a qualitative and exploratory study design in which 25 participants were interviewed which had experience conducting research in the field of HM. Two informants were medical herbalists implementing the integration pilot in Ghana. Four important sites for study were discovered: Kwame Nkrumah University of Science and Technology, Kumasi Centre for Plant Medicine Research, Mampong-Akuapem and two hospitals with piloted integration of herbal medicine and biomedicine. Interviews ranged from 19 to 49 min, were recorded (audio only) and transcribed by researchers. Framework analysis model was implemented in order to structure the responses which included: identifying a thematic framework; indexing, charting; and mapping and interpretation. Athematic analysis was created based on the notes of the interviews. Indexing involved assigning certain portions of the transcript to themes. Charting helped to visualize this information (using charts) on Excel. Finally, this data was organized to uncover potential obstacles and opportunities of the integration process and HM policy-making within Ghana.	Most participants have demonstrated that the proposed integration is positive in terms of homogenizing and standardizing medical care in Ghana. There are also potential job opportunities that may come up as a result (such as herbalists), who might not have found employment after completing their degree in HM. With increased HM knowledge, perceptions and awareness in Ghana, the potential to conduct integrated medicine research in hospitals is growing. This combination can now permit efficacious service delivery by inhibiting the practice of fake doctors, especially ones who are distributing fake HMs. With increased financial funding from the government, the country can now build more HM hospitals. These places can then serve as research departments for conducting research studies on HM for various types of diseases.	Deficiency of administrative procedures and precise articulation of government policy on the new integration could be a limitation to efficient implementation. There have been instances of delay in the integration process from the government side, with reduced initiative to jumpstart integration at peripheral pilot hospitals. The integration is mostly occurring in urban areas and not rural places, even though they require these kinds of therapies the most. The National Health Insurance Scheme does not encompass all the HMs being used by different units so sometimes there are cases that patients must pay out of pocket to access these in comparison to those who obtained treatment from physicians offering conventional care. Over time, there has been an increase in the lack of trust between healthcare professionals in the two units: biomedicine and HM, with many not being on the same page about the integration process. With a lack of funding, there is a hardship in herbal medicine registration and improving existing frameworks. Lastly, due to a lack of awareness about the integration and training on HM at hospitals, it has severely restricted the integration process by reducing the approved herbalists proficient in HM training.
da Justa Neves et al. 2015 [34]	Information reported by the US Food and Drug Administration was utilized which classified dietary supplements into the following main categories: sexual enhancement, weight loss, and muscle building. Sexual enhancement products included sildenafil, tadalafil, vardenafil and their equivalents, alone or in combination. Weight loss products majorly contained sibutramine. Muscle building products contained either an anabolic steroid (not mentioned) or an aromatase inhibitor. Legislation data related to supplements in Brazil, European Union and the United States were also analyzed.	Normally, in the European Union, information and notifications are collected through a database known as Rapid Alert System for Food and Feed. The research was conducted using ANVISA database where the researchers looked for technical reports about dietary supplements with prescriptions. Any information about those product samples that were seized by the Federal Police force known as Brazilian Federal Police Department was accumulated from its Criminalistics System (SisCrim).	The legislation about Brazilian supplements was examined using the ANVISA database, as well as the European Union using the Rapid Alert System for Food and Feed and the United States using the Food and Drug Administration. This was to garner enough info to make comments on the global condition of supplement contamination, and examine those substances that were seized by the Brazilian Federal Police Department. The legal standards for the three countries were also assessed: dietary Supplement Health Education Act for the United States, Regulation (EC) 178/2002—Council of Europe for the European Union, and the Decree-Law 986/1969 for Brazil.	Consumer markets have had a positive reaction to regulations whenever a new product is released. Consumers are also more inclined to believe that since HMs are sometimes categorized as “food”, they have little to no side-effects and the adverse effects, if any, are rarely displayed on product labels. With improved regulations, it will minimize the amount of adulterated dietary supplements. With current regulations that are not as stringent, has been shown to lead to greater adulteration cases. Increased strictness is a point of emphasis as currently several products are sold as dietary supplements without any comprehensive research being conducted beforehand. This especially is an area of concern for the United States which currently has rigorous lab and data accuracy measures compared to the European Union and Brazil.	Presence of varied universal regulatory assessments and policies. This causes a restriction in regard to judging and implementing a robust HM policy system in Brazil. Thereby, some products that are “harmless” could be deemed as irregular or unsafe and will be excluded from accepted HMs in novel policies (implying decreased variety for consumers).
Carvalho et al. 2014 [26]	The study assessed various documents obtained from the World Health Organization. It also identified the requirements for HM registration in different countries including those in Europe as well as, Australia, Mexico, and Canada. The following document from ANVISA was also analyzed: “Guide for the conduction of non-clinical toxicology studies and safety pharmacology required for the development of medicines.”. Governmental documents were analyzed like chromatographic profiles of HM species, usage restrictions, and daily dose.	Official documents were gathered from the World Health Organization along with papers elucidating HM registration processes and directives in international legislations including the European Community, Australia, Mexico, and Canada. The ANVISA database was also used for information gathering.	ANVISA created a survey for use that was adapted from the World Health Organization along with papers elucidating HM registration processes and directives in international legislations including countries in Europe, as well as, Australia, Mexico, and Canada. Rules and guidelines of these countries were examined, and listed down the main arguments.	Brazil policies and public opinions have both favored the research, development and implementation of the regulatory framework of HM. The government has also incentivized various research departments and institutions for performing scientific research with medicinal plants, emphasizing the nation’s biodiversity. Hence, ANVISA has made great efforts and is always on the lookout for improving current frameworks, prioritizing being in-sync with international drug regulatory authorities. This will do wonders in promoting safe access and rational consumption of HM products. Many legislative bodies are simultaneously working on revisions to Brazilian regulatory systems. These new frameworks will enhance regulator measures in lieu of appropriate technology transformations, permitting the expansion of such products to the general population.	Cumbersome and time-consuming research and policy approval process discourages organizations and academic institutions from performing research. This is especially the case when a number of permissions are to be acquired from ethics committees before beginning the research trials. Sometimes, these approvals are required before each of the phases I, II, and III to protect the population undergoing the study.
Awodele et al. 2014 [7]	In this study, actors such as health professionals and key stakeholders (directors, policymakers, etc.) were identified from the following institutions: Federal Ministry of Health (Herbal Medicine Division), The National Agency for Food, Drugs, Administration and Control (Herbal Medicine and Pharmacovigilance Division), The National Association of Nigeria Traditional Medicine Practitioners and the Nigerian Institute of Pharmaceutical Research and Development.	An organized questionnaire using the World Health Organization’s official documents was formulated to aggregate perspectives of relevant stakeholders in Nigeria working in policy-making or regulation within the field of HM. The questionnaire contains various sub-sections such as; National policy on Traditional Medicine, Law and Regulation on Herbal Medicine, Safety of Herbal Medicine, National Programme and National Research Institute on Traditional Medicine, Registration and Manufacture of Herbal medicine.	An organized questionnaire was provided to relevant stakeholders in Nigeria working in HM policy-making or regulation within the field of HM. The data obtained from the questionnaire were analyzed using simple percentages to document the perspectives and answers of the stakeholders.	Health authorities are now eyeing both the safety and effectiveness of HMs as important health concerns for the general public. This suggests that the vitality of instilling robust policies for the regulation of traditional medicine have become more important than ever. A fair and equitable framework development has been pushed for by the government that is reasonably regulated to ensure quality control requirements are met. In recent times, there has been a rise in collaboration with tertiary stakeholders and establishment of workshops to educate the public about the access and safe use of HMs. There is a critical need to ensure Nigeria contains some of the effective and high-quality medications as many respondents (68.7%) feel that the country lacks appropriate regulatory regimens.	In the study, about 62.5% of participants felt that limited research information could be a deterrent. About 31.3% see lack of professionals and knowledge within the health authorities as a challenge. The respondents seemed to have below average apprehension of the regulations as lower than 38% of respondents were aware of the National Agency for Food and Drug Administration and Control (Scientific Committee on Verification of Herbal Medicine).
Sahoo et al. 2013 [24]	150 Indian herbal drug companies that are involved in one or more activities of raw material collection/ trading, extract preparation, drug manufacturing, or contract manufacturing only. Responses were collected by email, telephone, and personal interviews from June 2009 to August 2010. Responses were mainly collected from the directors/proprietors/research and development heads of the companies as applicable.	A questionnaire-based survey was developed. It comprised 24 points and was administered to Indian HM firms involved in one or more of raw material obtainment/trading, extract preparation, drug or contract manufacturing only. The questionnaire constituted three different stages. The first component asked details about the representatives and information on general aspects of the company . The second module gathered information pertaining to any challenges faced during any of the following stages of raw material acquisition to marketing of the products and quality control procedures. The final part evaluated the export and import processes (submission requirements, review of timelines and its appropriateness, and support required from the government). Details from other organizations displaying good notions about Indian manufacturing companies were also noted. Data in the context of India’s GMP regulation and legislative procedures for HMs were also examined.	A questionnaire was used to gather information on challenges faced during production, commercialization, and marketing approval of HMs in India and foreign countries. These responses included opinions from 150 different companies and were collected via email, telephone, and in-person interviews from June 2009 to August 2010. The data obtained were examined by the researchers and key takeaways were drawn.	India’s governmental authorities have done their share of work by assembling organizations, events, workshops, exhibitions, research opportunities all while providing technical and financial support to universities. These initiatives can facilitate the scientific approval of Indian HM frameworks globally. However, inadequate safety control measures in different areas of manufacturing remains an important concern as over 60% of participants indicated that additional quality control proceedings should be developed. Devising harmonized regulations, and defined timelines, will assist in reducing the number of errors and outliers in the context of state-licensing laws. Not only will this remedy problems like product contamination, but also resolve issues such as substitution.	The survey conducted unveiled how regulatory compliance remains the number one challenge for exporters. Like many other countries, the inconsistency in GMP is outlined as the primary impediments for Indian manufacturers. Conflicting regulatory procedures worldwide and application delays were another area of concern. Worldwide variability in herbal medicine approval process: loose laws in India, whereas in the European Union it needs to successfully pass through a full regimen, requiring exhaustive safety and efficacy data. In addition, there exists a scarcity of herbal practitioners. Drug manufacturers are not able to keep up with increasing standards for herbal medicinal products. There also exists inadequate research and development, and slow pace of modernization.
Walji et al. 2013 [29]	15 key informants were interviewed, including government representatives (*n* = 5), industry (*n* = 9) members from large and small companies and consumer groups (*n* = 1), with observation of a government consultation meeting. Analysis (*n* = 38) of public documents about legal and policy information with regard to NHP regulations.	Public documents on legal and policy information about NHP regulations were reviewed in their analysis. Semi-structured interviews with key informants were audio recorded and transcribed.	Semi-structured interviews with key informants were conducted from 2009 to 2010, audio recorded and transcribed, as well as observation at a consultation meeting with members of the industry. Documents of scientific, government, and grey literature for analysis were found through online search and referral from key informants. There were two researchers who coded the data collected from content analysis of policy documents and interviews, organizing the information in emergent themes within a regulatory context.	Industry key informants support the NHP regulations, agreeing that strict and rigorous standards of manufacturing and labeling were needed. Clear communication and consultations sessions during the regulatory process through feedback channels and increased interactions between stakeholders involved in refining the product categories Clarification of definitions and pre-clearing information in product licensing forms support staff of companies through these applications as well as allow regulatory assessors to more efficiently review applications.	The Canadian government has had difficulty classifying NHPs, as the field of NHPs are labeled differently internationally, as well as difficulty keeping a set of rules of formulation and categorization consistent across the wide class of NHP products. Regulations regarding the level of evidence needed to support the safety and efficacy of products are in disagreement between the government and industry members. External groups have lobbied for strict compliance with regulation, creating conflict and tension. Classification of NHPs as its own unique category is ambiguous and blurred, creating confusion. Industry workers are concerned over possible delays in the authorization process.
Alameddine et al. 2011 [27]	Stakeholders of the complementary and alternative medicine market in Lebanon were interviewed (*n* = 16), which included decision-makers, representatives of professional associations, academic researchers from the faculties of agriculture, nutrition and pharmacy, owners or companies of complementary and alternative medicine product importers, policymakers from the Ministry of Health and Ministry of Trade, and a media representative who is familiar with the field.	Data were collected from semi-structured interviews with key informants, using ten questions. They were also asked about any relevant documents and additional stakeholders that could contribute to this study. Field notes were taken and analyzed, and along with the interview tapes and transcripts, recurring issues and data patterns were identified.	Sixteen semi-structured interviews with stakeholders of the Lebanese complementary and alternative medicine market, and these interviews were transcribed. The scripts were analyzed for any emerging themes, identified through recurring issues and patterns that were arisen from the data.	There was widespread agreement from most stakeholders about the existence of poor regulation and lack of knowledge of safe complementary and alternative medicine consumption among the public and those involved in the Lebanese complementary and alternative medicine sector, due to the lack of policies and procedures, as well as issues with compliance to existing regulations. There seemed to be support from stakeholders to adopt mixed and compulsory policy instruments by improving public and provider awareness on the safety of complementary and alternative medicine products, paired with strict governmental regulation of the complementary and alternative medicine market.	According to stakeholders, current regulations lack effectiveness due to complementary and alternative medicine importers’ poor adherence to existing rules and guidelines paired with loopholes in current regulations that restrict complementary and alternative medicine products’ entry through other venues. Other barriers included the fragmented healthcare systems and the lack of cooperation and coordination between the different ministries of health.
Moss et al. 2007 [31]	Canadian complementary and alternative medicine group leaders (Western herbalism (*n* = 9), naturopathy (*n* = 10), traditional Chinese medicine (*n* = 8), and homeopathy (*n* = 10)), were interviewed. This included presidents, chairs, heads or directors who were selected from a list of associations, schools and journals. A Green Paper (government document that details specific issues, outlining possible courses of action in terms regarding policy) was published in the Ontario Herbalists' Association quarterly journal and analyzed in this study.	Interviews with key informants were conducted, with questions about the participants’ perceptions of the NHP regulations. The interview transcript was obtained and coded by at least two investigators. Documents including the Green Paper were also analyzed.	Formal leaders were chosen from a list of associations, schools and journals in order to get participants from various different segments. Interviews with the leaders were tape recorded, transcribed, and inputted into a qualitative computer software program to be analyzed. Textual data, such as the Green Paper was analyzed based on the Western herbalists’ thoughts about the NHP regulations.	Development of a national standard of practice of herbal medicine by the national council could regulate practice standards and improve group cohesion. Western herbalists may be in a better position to implement the other strategies—improving practise standards, education standards, and engaging in peer-reviewed research—and thereby advance their professionalization by fostering group cohesion through a national council.	Based on the interviews with Western herbalist leaders, there existed opposition to the standardization of NHPs used in their practice; they expressing their expectation of implemented regulations to properly reflect the risks involved with NHPs, which they felt did not happen. The new NHP Regulations’ requirements would be too rigorous for small-scale manufacturers, lowering compliance and causing many Western herbalists to go out of business.
Laeeque et al. 2006 [6]	Sixty-seven out of the 364 NHP businesses in Canada met the eligibility criteria. Presidents/owners, managers, and any quality assurance personnel from 20 of the 67 companies were interviewed. Eleven of these companies were based in Ontario, 3 companies were from British Columbia, and 3 companies were from Quebec.	Semi-structured interviews were conducted in-person or through the telephone, audiotaped and transcribed verbatim. Each respondent was mailed a survey prior to the interview to collect information on the size of the company, the number of NHPs products they sell, and details about the glucosamine/chondroitin in these products.	This qualitative study is an applied ethnography, interviewing key informants regarding satisfaction and compliance with the Regulations. Interviews were tape recorded and transcribed. Content analysis on the data was independently coded, and emerging themes were determined through team meetings held incrementally after 3–4 interviews.	All employees who were interviewed agreed with the goal of ensuring the safety, quality, and efficacy of NHPs, stated by the Natural Health Products Directorate of Health Canada. Many informants thought that the regulations could improve the public's confidence in NHPs, which will help the NHP industry. Specifically, strict regulations can cause marginal firms to exit the markets, in an attempt to protect consumers from these firms and their products. Submissions of producing licensing applications suggest generally positive views of regulations and better compliance, which the majority of the companies from the sample were doing.	Interviews revealed some disappointment and frustration with the slow pace of progress, specifically with the implementation stage. Some small and medium-sized enterprises representatives felt that the control of these regulations were at the same level as conventional drug regulations in Canada. There was also discontentment towards the government’s consideration of NHPs as a subset category of drugs as they were perceived by interviewees to have different volumes of sale, price mark-ups, and risks involved. Due to regulations, smaller companies may struggle with affordability and undeliberate failure to comply, and beneficial products could be lost. Many participants were worried that the regulations will be too strict, be limited in focus for one part of the NHP industry, and be reactive as opposed to proactive. Lack of compliance could result from a lack of knowledge on regulations from employees and owners.
Moss et al. 2006 [30]	Formal and informal leaders of 4 complementary and alternative medicine practitioner groups in Canada that use natural products as part of their core practice (*n* = 37) were interviewed, which included Western herbalism (*n* = 9), naturopathy (*n* = 10), traditional Chinese medicine (*n* = 8), and homeopathy (*n* = 10).	Semi-structured qualitative interviews with 37 Canadian leaders of four complementary and alternative medicine practitioner groups were conducted in the Fall of 2004. Content analysis was done by identifying themes, sub-themes, and any existing relationships.	This applied ethnographic study included data collected from qualitative interviews. The interviews were transcribed and analyzed independently by at least two investigators. All transcriptions were inputted into a qualitative computer software program, and themes were identified and reported.	Naturopaths have the ability to prescribe from a list in four Canadian provinces, seemingly legitimizing their knowledge claims. The public, without proper practitioner guidance and lack of knowledge, could be at risk of misusing herbs. Different health care practitioners with proper training should have access to high-risk products. Western herbalists expressed their view of pharmacists as inappropriate gate-keepers of NHPs with their inadequate knowledge of herbs, and TCM leaders were concerned that NHPs were not labeled correctly. Moreover, naturopaths firmly believed that NHP regulations will not harm their core practices, but rather strengthen their role within the healthcare system. Western herbalist leaders did not believe that NHP regulations would encourage self-medication herb use.	Regulation of naturopaths across countries is inconsistent, resulting in inefficient cooperation with naturopaths who are not regulated. There exists conflicting mindsets between Western herbalists and naturopaths in that herbalists want the public to be self-sufficient. There is also a fear from TCM leaders that the Natural Health Products Directorate will over-regulate NHPs, which may potentially eliminate certain supplements. Naturopaths, TCM practitioners, homeopaths, and Western herbalists seemed to disfavor the implementation of regulatory laws due to possible decreased access to products. It is difficult to define complementary and alternative medicine due to differing beliefs, principles or practices between complementary and alternative medicine practitioner groups.

Abbreviations: *ANVISA* National Health Surveillance Agency of Brazil,* DHS* dietary and herbal supplement,* GMP* good manufacturing practices,* HM* herbal medicine,* INP* indigenous natural product,* NHP* natural health product,* TCM* traditional Chinese medicine

### Finding from thematic analysis

The regulation of DHSs was explored through the following themes representing a facilitator or barrier to DHS regulation: (1) increased financial and human resources encouraging knowledge expansion as a facilitator to DHS regulation; (2) variances in DHS classification and regulatory requirements across countries as a barrier to DHS regulation; and (3) collaboration between various stakeholders (experts, policymakers, representatives of regulatory bodies, product companies and research institutions) facilitating DHS regulation.

### Theme 1: increased financial and human resources encouraging knowledge expansion as a facilitator to DHS regulation

Financial initiatives to encourage research on DHSs have positively impacted their regulation in eight countries, especially with new regulatory frameworks now requiring rigorous scientific testing and sufficient research data before the registration and marketing of these products [24]. HM researchers in Ghana did not have adequate funding to conduct clinical trials and measure safety and efficacy of HMs. Due to the lack of trial data, they struggled to register herbal supplements for marketing [25]. Conversely, in Brazil, the National Policy on Medicinal Plants and Herbal Medicine has encouraged research in medicinal plants through financial incentive programs organized by the Ministry of Health and other governmental agencies, such as the National Committee of Medicinal Plants and Herbal Medicines, that work to support the harmonization of the Brazilian market. Regulatory authorities are then able to refer to one unique set of information on a herbal substance when evaluating marketing applications for HMs [26]. It was also noted that more than 58% of herbal medicine research programs were established solely due to incentives offered by local authorities and the federal government of Brazil [26]. Similarly, the government of India has organized exhibitions to promote knowledge exchange between scholars in the field of HM and provided technical and financial support to universities and institutions specializing in HM research [24]. The government’s establishment of the Indo-US Centre for Research on Indian Systems of Medicine has also promoted collaboration on research between traditional medicine and Ayurveda practitioners in India and HM researchers in the United States. Encouraging this integration of evidence-based traditional medicine methodologies from the United States with traditional herbal medicine systems, such as Ayurveda, Siddha, and Unani, in India has led to the development of training workshops involving both countries [24]. Moreover, evidence-based submissions of safety and efficacy studies on herbal supplements in India have become increasingly important for receiving marketing approval, and as such, there has been an increasing necessity to focus on scientific and technological advances within the field of HM [24]. Therefore, the implementation of such programs helped to ensure that India acquired adequate scientific approval of its HM frameworks in other countries [24].

In addition to research, there has been a need for greater financial support to hire sufficient staff and train them to implement HM policies. The National Health Regulatory Authority of Bahrain, for example, has benefited from its high number of scientific reviewers due to their familiarity with policy implementation, which does not require additional training. This abundance of reviewers also facilitated the rapid implementation of HM classification guidelines [18]. On the other hand, Kuwait’s lack of properly trained reviewers and severe staff shortages has prompted several key officials from its drug regulatory authorities to propose an increase in the number of reviewers involved in the HM registration process, including herbal specialists who are knowledgeable in HM science, in order to resolve any confusions that may arise in policy-making discussions [18]. Similarly, Lebanese health officials lacked a central, federally run laboratory to conduct robust safety and efficacy testing before complementary and alternative medicine products were introduced to the market because they do not have adequate human resources at their Ministry of Health headquarters. As a result, the current regulatory body has struggled to efficiently regulate complementary and alternative medicine products effectively without the adequate expertise available to analyze and monitor them [27].

### Theme 2: variances in DHS classification and regulatory requirements across countries as a barrier to DHS regulation

The differences in classification and existing regulations of DHS products have caused ambiguities, making it difficult to develop and implement regulatory policies. For instance, Brazilian policymakers and government officials have been unable to homogenize the definition of HM across international drug regulatory authorities and thus lacked clarity when deciding under which category to register the products. This allowed different HMs with the same active ingredients and characteristics to be registered in more than one department, creating inconsistencies and duplications in product registration [18].

The Kuwait Drug and Food Control and Authority regulatory system allocates HM supplements under the “Herbal”, the “Dietary Supplement”, or the “Unclassified” departments based on their composition; however, the absence of clear classification guidelines has led to inconsistent registration of different HM products with identical active ingredients [28]. Additionally, HMs that were registered under the unclassified unit as dietary supplements or as functional food under the food supplements unit required fewer and less stringent requirements for registration, and thus inappropriately circumvented rigorous safety and efficacy evaluations. Since Kuwait has frequently imported HMs from the United Kingdom, Germany, the United States, the United Arab Emirates, and the Kingdom of Bahrain, variances in herbal supplement categorizations have made it difficult for the Kuwait Drug and Food Administration to adopt existing classifications into their drug regulatory authority system and subsequently regulate the same products in their own country [28]. Therefore, a standardized definition may help to ensure that all HMs imported from other countries undergo the most appropriate conformity assessment before entering the Kuwaiti market to become regulated under one of five separate registration units (pharmaceutical, herbal, veterinary, unclassified, cosmetics and food supplements unit) [28].

Differences in regulatory requirements between herbal medicine approval procedures and submission requirements of India, the United States, and the European Union have presented consumer risks. Under the Drug and Cosmetics Act of 1940 in India, for instance, traditional HMs with a long history of use are assumed to be safe, and thus safety or efficacy data on their products is not necessary for marketing approval. Indian manufacturers have sold the majority of these HM products in the United States as dietary supplements because they do not require approval prior to producing or selling DHSs, and such categorization does not require scientific evidence of safety nor efficacy under the Dietary Supplement Health and Education Act of 1994 [24]. This has led to subsequent delays in Indian HM application processes requiring preclinical safety data (including toxicologic and pharmacologic test data) due to the lack of pharmacopeia harmonization that would better support the registration of drugs including HMs across countries. For example, manufacturers in the United States have struggled to market their HM products in India because unlike the United States Pharmacopoeia, the Indian Pharmacopoeia does not have a separate category for dietary supplements. As a result, government regulators who review drug applications are constantly faced with the challenge of the lack of consistency with pharmacopeia standards across different countries [24]. Small and medium-sized manufacturers also faced commercialization issues as they were burdened with the need to perform product analyses based on different acceptance criteria in order to satisfy pharmacopoeial requirements that vary across regions. Therefore, standardization of accepted quality parameters for HM products and ingredients could aid in providing standards for their quality control [24].

In Canada, NHPs were not previously categorized distinctly from food and drugs, resulting in confusion surrounding where certain products should fit. Having NHPs classified as a separate category has facilitated the implementation of regulations, which have since been continuously revised [29, 30]. Overall, the adoption of a synchronized regulatory framework and a unified definition of NHPs in Canada, accomplished through consultations with industry representatives and policymakers, has facilitated bilateral communication to allow for the refinement of existing regulations [29]. The inclusion of multiple perspectives in the discussion also aided in clearly defining product categories and could serve as a resource for other jurisdictions planning to implement NHP regulation as a part of their established regulatory frameworks [29].

### Theme 3: collaboration between various stakeholders (experts, policymakers, representatives of regulatory bodies, product companies, and research institutions) facilitating DHS regulation

Collaboration among stakeholder groups through committee meetings, workshops, and the creation of expert committees may help in developing effective regulatory policies for DHSs. The Nigerian government concluded that there was a need for collaboration with global partners, specifically the World Health Organization that has developed programs to foster information sharing about regulatory issues such as the lack of research data, appropriate mechanisms for the control of HM, expertise within the health authorities and control agencies, and monitoring of consumer safety at international workshops. In this way, Nigeria could build its national capacity on establishing HM regulations [7].

In a study conducted in Kuwait, managers working in the registration of HMs and scientific reviewers performing quality assessments of HMs found that the increased cooperation between government officials in the National Health Regulatory Authority and the Saudi Food and Drug Authority was crucial in addressing the need to introduce an HM classification policy. Committee meetings between Kuwaiti government officials and external experts were seen as opportunities for sharing ideas, knowledge, and expertise. Consequently, clear objectives and realistic timelines for developing effective regulatory policies and guidelines for the classification of HM products were established [18]. Moreover, regular meetings between scientific reviewers and senior managers involved in the HM product registration process allowed the two stakeholder groups to decide whether to register particular products under the traditional herbal medicine registration pathway, resulting in a smoother classification process [18].

In Namibia, the forum Devil’s Claw Working Group was established to facilitate policy development for devil’s claw (*Harpagophytum* spp.). In collaboration with the Ministry of Environment and Tourism-Directorate of Natural Resource Management, this forum has hosted international consultations and workshops for the review and harmonization of devil’s claw’s regulatory policies [9]. Working groups in the Southern Africa region were also established for the facilitation of information exchange and joint learning practices of INPs between South Africa, Botswana, and Namibia [9]. Likewise, Nigeria has recognized the importance of having expert committees review HMs and has established a Scientific Committee on Verification of Herbal Medicine to ensure the safety, efficacy, and quality of these products [7]. In creating this committee, the Federal Ministry of Health focused on increasing collaboration among stakeholders and ensuring effective implementation of Nigerian regulations for the safe use of HMs. Similarly, in Canada, communication and consultation sessions are held annually by the Natural Health Product Directorate Advisory Committee to allow for the exchange of feedback regarding NHP regulations between stakeholder groups and the subsequent refinement of policies and product categories [29, 31].

Furthermore, the lack of contact between Brazilian HM product companies and national research centers resulted in insufficient university research and information on the development or licensing of HMs before receiving marketing approval [32]. Therefore, the National Health Surveillance Agency of Brazil (ANVISA), which oversees the licensing and health surveillance of HM products, has recognized the importance of knowledge mobilization and active collaboration between the HM researchers and regulators, and thus has engaged in various cooperative initiatives. Most notably, the International Pharmaceutical Regulators Forum encourages coordination between countries such as the United States, Canada, and India for the exchange of information gathered from research investigations, which may help to strengthen existing research on HM regulations in other countries [33]. ANVISA has even proposed organizing regular meetings with the Food and Drug Administration (FDA) to share information on regulatory policies regularly [33, 34].

## Discussion

### Summary of findings

The purpose of this scoping review was to identify the facilitators and barriers to DHS regulations internationally. Our systematic search yielded 15 articles that met our eligibility criteria and were included for data extraction. Our analysis identified three main themes: (1) increased financial and human resources encouraging knowledge expansion as a facilitator to DHS regulation; (2) variances in DHS classification and regulatory requirements across countries as a barrier to DHS regulation; and (3) collaboration between various stakeholders (experts, policymakers, representatives of regulatory bodies, product companies and research institutions) facilitating DHS regulation. To our knowledge, this is the first study to systematically evaluate and summarize facilitators and barriers to DHS regulatory frameworks globally; our findings may provide policymakers, healthcare professionals, and other stakeholders with insight into what specific factors affect DHS regulation.

### Unique challenges associated with DHS regulation

DHSs have generally lacked well-developed regulatory frameworks when compared to that of pharmaceuticals and medical devices, resulting in a variety of unique challenges. In the United States, DHSs can be sold and marketed around the world without providing the FDA with evidence of the product’s safety and efficacy, as mandated by the Dietary Supplement Health and Education Act [35]. A meta-analysis of 26 high-quality systematic reviews reported that DHSs were commonly contaminated with dust, pollen, and/or toxic heavy metals that could cause severe adverse effects, such as multi-organ failure and lead or mercury poisoning [36]. Furthermore, safety risks of raw herbal materials require attention as western countries such as Australia lack regulations for their imported materials, especially from China [37]. Specifically, the regulation of restricted and contaminated substances conducted by Customs and Quarantine control, a department managed by the Australian Department of Home Affairs, has insufficiently conducted appropriate quality control procedures that should be performed to detect any contamination or pollution of these substances, which may include the use of mass spectrometry and chemical fingerprinting [38]. Moreover, due to the lack of a clear classification system in Kuwait, some DHSs have been registered under the unclassified unit as dietary supplements, which has fewer stringent requirements for registration. As a result, some products are marketed inappropriately, presenting health and safety issues to the public [7]. Additionally, although guidelines have been drafted for the preclinical safety evaluations of Ayurveda, Siddha, and Unani within the Indian DHS registration system, there are currently no regulatory policies for the standardization of herbal preparation and marker-based identification of active components, which could lead to adulteration, misidentification, mislabelling, and contamination of DHSs [24, 39]. In contrast, there have been initiatives to improve existing regulations and to help mitigate these safety concerns elsewhere. For example, the Committee on Herbal Medicinal Products in the European Union was established to harmonize the regulation of HMs and facilitate its marketing [40]. The European Pharmacopeia was developed to provide a system of complete technical standards and requirements for the regulation and application of HMs in the European Union market, creating a standard for the safety and efficacy evaluation of DHSs [41].

In addition to the inherently complex nature of DHSs, adverse effects reported by consumers have been linked to deviations from good manufacturing practices (GMP), intentional substitutions and adulterations from the use of cheaper components, and improper preparations and dosages [42, 43]. In an effort to raise standards of safety and quality, inspections of DHS manufacturing facilities are being conducted under the GMP, which is overseen by regulatory agencies such as the Center for Drug Evaluation and Research in the United Kingdom, United States, Canada, the European Union, China, and India [43]. It is difficult for manufacturing companies to correctly identify plant parts and starting materials of DHSs when complying with GMP due to the high phenotypic variation between similar plant products, unidentifiable plant extracts, and the lack of highly trained professionals in plant taxonomy that leads to mistaking one herb for another due to their similar appearances or incorrect labeling [44, 45]. Therefore, the World Health Organization has recommended implementing national quality specification measures and standards, GMP, labeling, and licensing schemes for manufacturing in countries where DHSs are regulated [45]. Furthermore, the classification of herbal medications as dietary supplements under the United States Dietary Supplement Health and Education Act allowed DHSs to be marketed without providing proof of safety and efficacy, instead shifting the responsibility of blocking the marketing of unsafe dietary supplements to the FDA which remains understaffed and underfunded to fully enforce these rules [46]. These relaxed regulations have benefited manufacturers, as being subject to many legal requirements is often challenging and counterproductive to the production process [47]. There has even been a divide between regulators and manufacturers with regard to quality testing of DHSs, as manufacturers are hesitant about requiring stricter analytical methods for DHS ingredients due to the significant expertise and monetary costs that are involved [35]. In fact, conducting clinical trials to demonstrate a new pharmaceutical’s safety and efficacy in the United States was estimated to cost $500 million, which is uneconomical for the private industry of DHSs [48]. Namely, unlike pharmaceuticals, DHSs cannot be patented, causing manufacturers to face price pressure from their competitors and thus lack the profits to fund such costly clinical trials [49].

### Future areas of research

The differences in DHS classification guidelines and definitions between drug regulatory authorities is one of the primary factors that have made it difficult for countries such as Brazil and Kuwait to regulate these products consistently over time. For instance, DHSs with the same ingredients have been placed under multiple categories that have different requirements of safety and efficacy evaluation due to a variety of registration applications being processed for similar products [18]. Science-based quality standards, such as the up-to-date standards, may help to set appropriate quality attributes of DHSs between manufacturers, regulators, and other stakeholders when assessing their ingredients to ultimately homogenize the requirements, definition, and terminology of its categorization [50, 51]. Additionally, emerging technologies such as DNA barcoding and next-generation sequencing can help to identify the origin of products, discriminate between similar species, and ensure reproducible results, thus improving research to establish a common reference standard between agencies [52]. Surveillance systems, such as China’s Adverse Drug Reaction Monitoring System and the FDA’s MedWatch program, have also provided important information regarding adverse events associated with DHSs [52]. Further to this, 4 out of the 11 countries in South-East Asia have established national systems for monitoring the safety of DHSs. Hence, it has been suggested that a global surveillance system be established to assist with the identification of product origin and to communicate precise product characteristics [43]. The establishment of national safety monitoring programs for DHSs may facilitate the collection and analysis of reports of adverse effects from consumers of these products and subsequently aid in informing regulatory authorities of the appropriate safety and efficacy requirements.

### Strengths and limitations

A notable strength of this study includes the use of a systematic search strategy to identify the studies included in this review. Interpretation of these findings was strengthened by the fact that two authors (MK and AS) independently screened, extracted data, summarized findings, and met with the supervising author (JYN) to resolve any discrepancies. Limitations of this review include the exclusion of non-English language articles, potentially omitting studies that emerge from non-English speaking countries. Furthermore, a small number of articles were irretrievable, despite receiving assistance from our university librarian in placing interlibrary loan requests. We also did not include a search of the grey literature and acknowledge that this review may not necessarily capture industry researchers’ evaluations of global DHS regulatory frameworks; however, this is justified considering the parameters of our research question, which was to assess DHS regulations specifically and identify only research in the peer-reviewed literature.

## Conclusion

The present scoping review systematically searched the literature to identify facilitators and barriers to DHS regulation across different countries while also highlighting several important themes. Our findings suggest that the adoption of a harmonized regulatory system could help to ensure that products are evaluated with the most appropriate conformity assessments, and that government financial and human resource incentives could promote the conducting of more ethically sound international DHS research. Furthermore, a greater degree of collaboration between stakeholders could encourage increased resource mobilization, the development of knowledge exchange initiatives, and the establishment of feedback opportunities to improve regulations. We also highlight that safety assessments of DHSs continue to be inadequate, and emerging technologies could potentially play a significant role in establishing common reference standards of herbal materials and products between regulatory agencies. Finally, we believe that regulatory harmonization, increased scientific research, and greater collaboration could improve regulations globally by ensuring appropriate categorization and safe application of DHSs.

## Data Availability

All relevant data are included in this manuscript.

## Abbreviations

ANVISA: National Health Surveillance Agency of Brazil
DHS: Dietary and herbal supplement
FDA: Federal Drug Authority
GMP: Good manufacturing practices
HM: Herbal medicine
INP: Indigenous natural product
NHP: Natural health product
T&CM: Traditional and complementary medicine
TCM: Traditional Chinese medicine
